# Porcine epidemic diarrhea virus: a model for coronavirus persistence and immune escape in intensive livestock systems

**DOI:** 10.1186/s40813-026-00520-6

**Published:** 2026-05-05

**Authors:** Leila Wogick, Sagar M. Goyal

**Affiliations:** Veterinary Diagnostic Laboratory and Veterinary Population Medicine Department, College of Veterinary Medicine, University of Minnesota, St. Paul, MN 55108 USA

**Keywords:** PEDV, Spike glycoprotein, Enteropathogenic coronavirus, Swine

## Abstract

Porcine epidemic diarrhea virus (PEDV) is an enteric alphacoronavirus that has shifted from a sporadic regional pathogen to a worldwide, persistent virus, despite years of vaccination, biosecurity measures, and monitoring. Since the early 1970s, PEDV has shown strong genetic flexibility, the ability to evade immunity, survive in the environment, and spread quickly through connected livestock networks. Major outbreaks in Asia, Europe, and North America, especially the 2013–2014 outbreak in the United States, have revealed key weaknesses in traditional control methods for enteric coronaviruses, especially those that depend on systemic immunization. In this review, we bring together over fifty years of PEDV research to look at how viral evolution, mucosal immune responses, and livestock production systems work together to keep the virus circulating. We point out that changes in the spike gene, including recombination and deletions, along with changes in other viral genes, affect disease severity, immune system recognition of the virus, and the inability of vaccines to provide protection. We also stress the key role of lactogenic immunity and the gut-mammary-secretory IgA system in protecting newborn animals, which helps explain why vaccine inoculations have not been effective in stopping the spread of enteric coronaviruses. By combining evidence from molecular, immune, epidemiological, and systems research, we suggest that PEDV is a strong example for studying how coronaviruses persist under immune pressure in managed animal groups. Using a One Health approach, this review encourages moving away from only reacting to outbreaks and instead focusing on ongoing, genome-based management of coronaviruses in livestock operations.

## Introduction

**Fig. 1:**
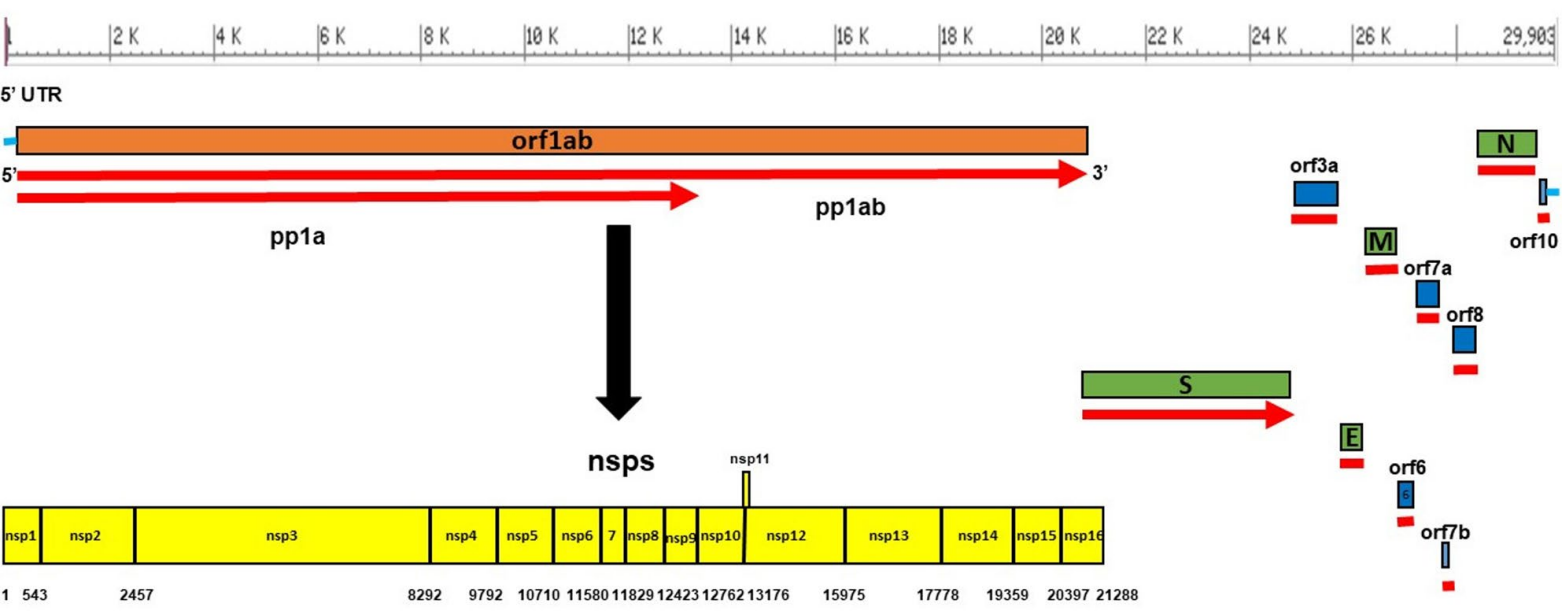
Coronavirus genome organization. Schematic representation of the coronavirus positive-sense RNA genome showing ORF1a/ORF1b encoding the replicase polyproteins and processing into nonstructural proteins (nsps), followed by the structural protein genes (S, E, M, N) and accessory open reading frames. Adapted from Helmy et al., Journal of Clinical Medicine (2020) under a Creative Commons Attribution (CC-BY) license [4]

Coronaviruses are characterized by a distinctive crown-like appearance created by large trimeric spike (S) glycoproteins projecting from the virion surface. The enveloped virus has a positive-sense, single-stranded RNA. Coronavirus particles are typically spherical to pleomorphic, measuring 100–150 nm in diameter, and consist of a lipid envelope embedding the spike (S), membrane (M), and envelope (E) proteins, which surround a ribonucleoprotein complex formed by genomic RNA bound to the nucleocapsid (N) protein. The coronavirus genome is among the largest of all RNA viruses (~ 26–32 kb) and follows a conserved organization, with ORF1a and ORF1b encoding the viral replicase polyproteins, followed by genes encoding structural and accessory proteins that govern viral entry, assembly, and host interactions [1–3] (Fig. 1).

Based on phylogenetic relationships and genome organization, coronaviruses are classified into four genera: Alphacoronavirus, Betacoronavirus, Gammacoronavirus, and Deltacoronavirus. Alphacoronaviruses and betacoronaviruses primarily infect mammals, whereas gammacoronaviruses and deltacoronaviruses predominantly infect birds, with occasional spillovers into mammalian hosts. Within the livestock populations, alphacoronaviruses are of particular importance due to their strong enteric tropism, high genetic plasticity, and capacity for sustained circulation under conditions that favor immune selection and environmental persistence [1, 3].

Swine are natural hosts to multiple enteric coronaviruses, including transmissible gastroenteritis virus (TGEV), porcine epidemic diarrhea virus (PEDV), porcine deltacoronavirus (PDCoV), and swine acute diarrhea syndrome coronavirus (SADS-CoV). These viruses share similar transmission routes, primarily fecal–oral spread, and target the intestinal epithelium, causing acute diarrhea, dehydration, and high mortality in neonatal piglets. The co-circulation of multiple coronaviruses within dense swine production systems creates opportunities for recombination, viral diversification, and the emergence of novel variants with altered pathogenic and antigenic properties [3, 5, 6].

Among these pathogens, porcine epidemic diarrhea virus (PEDV) has emerged as one of the most persistent and economically important enteric coronaviruses of swine. The PEDV was first recognized in Europe in the early 1970s as a distinct diarrheal disease and subsequently confirmed as a novel coronavirus through isolation and characterization of the prototype CV777 strain [7, 8]. Experimental infection studies demonstrated that PEDV selectively infects mature enterocytes of the small intestine, leading to severe villous atrophy, malabsorption, and dehydration, with mortality rates approaching 100% in naïve neonatal piglets [8–10].

**Fig. 2:**
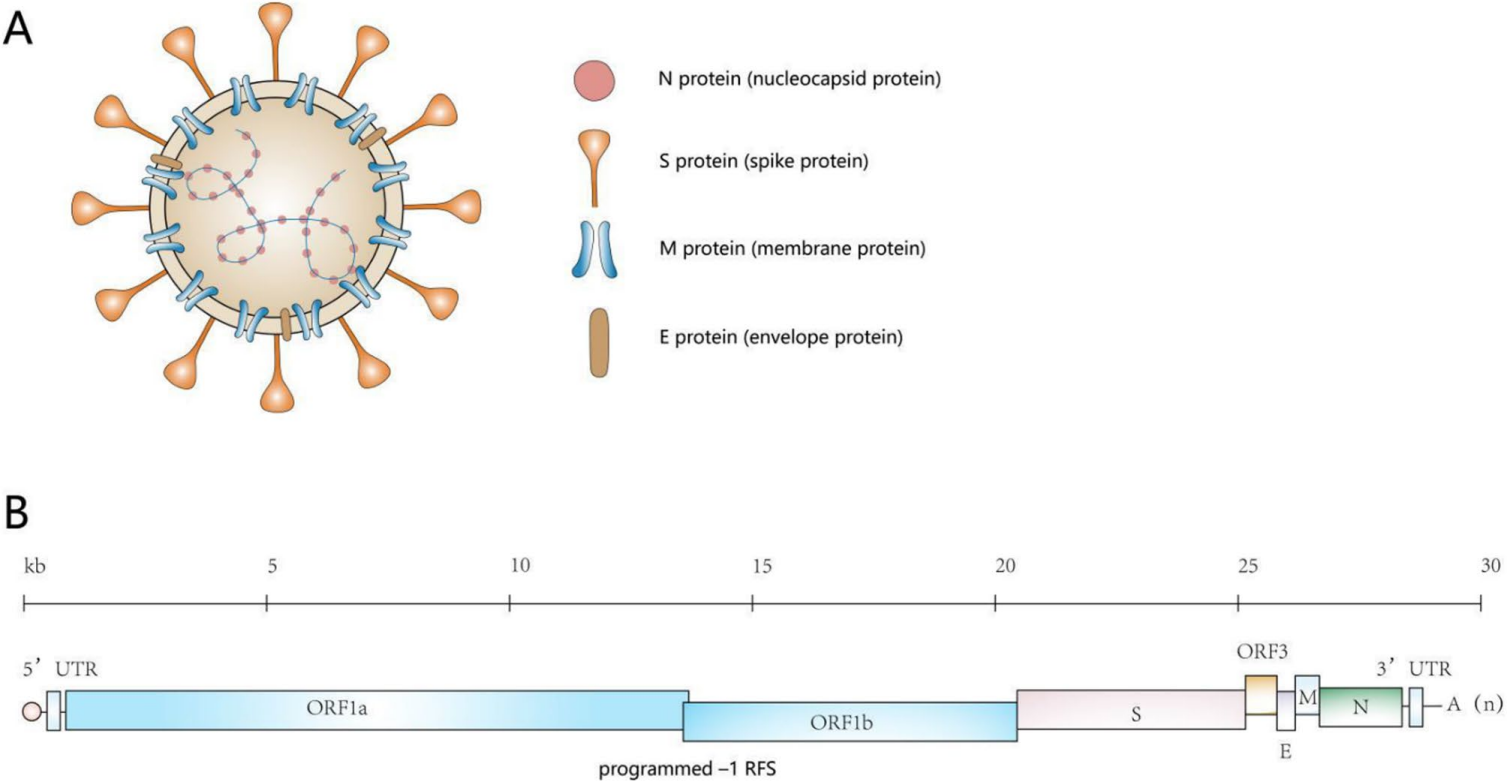
Genome organization and structural features of porcine epidemic diarrhea virus (PEDV). (**A**) Schematic representation of the PEDV virion showing genomic RNA bound to the nucleocapsid (N) protein and enclosed within a lipid envelope containing spike (S), membrane (M), and envelope (E) proteins. (**B**) Organization of the ~ 28 kb positive-sense RNA genome of PEDV, comprising ORF1a and ORF1b encoding the replicase polyproteins, followed by genes encoding the spike (S) glycoprotein, accessory ORF3 protein, and the structural proteins envelope (E), membrane (M), and nucleocapsid (N). Genetic variation in both spike and non-spike regions contributes to PEDV antigenic drift, altered virulence, and phenotypic diversity. Adapted from Lin et al., Viruses (2022), under a Creative Commons Attribution (CC BY 4.0) license [17]

PEDV possesses a large (~ 28 kb) positive-sense RNA genome encoding the replicase polyproteins, the spike glycoprotein, the accessory protein ORF3, and the structural proteins (envelope, membrane, and nucleocapsid proteins) [3, 11]. As in other coronaviruses, the spike glycoprotein mediates receptor binding and membrane fusion and serves as the primary target of neutralizing antibodies. Consequently, genetic changes in the spike protein, including point mutations, insertions, deletions, and recombination, have been major drivers of PEDV antigenic drift, altered virulence, and vaccine mismatch [3, 11–13]. Importantly, accumulating evidence demonstrates that changes outside the spike gene, such as truncations in ORF3 and deletions in the nucleocapsid gene, also influence viral replication efficiency, pathogenicity, and host immune responses. These findings suggest that PEDV evolution is influenced by multiple genomic determinants, rather than spike gene variation alone [3, 14–16] (Fig. 2).

The global significance of PEDV became particularly evident in 2013, when highly virulent strains were introduced into North America and spread rapidly across the continent [18, 19]. Within months, outbreaks were detected in more than 30 U.S. states, resulting in the loss of millions of piglets and economic damage exceeding $1 billion [18]. Genomic analyses revealed that North American epidemic strains were closely related to contemporary Asian lineages, underscoring the ability of enteric coronaviruses to disseminate between continents through global trade and interconnected production networks [19]. During this period, S-INDEL variants also emerged. Although these strains typically caused milder clinical disease, they complicated diagnostic interpretation and vaccine matching, illustrating how coronavirus genetic diversity undermines control efforts in endemic settings [12, 20, 21].

Following the North American epidemic, PEDV has continued to diversify through recombination, large spike-gene deletions, and co-circulation with other swine coronaviruses, including PDCoV and TGEV [5, 22–25]. These evolutionary processes are favored in pig populations with mixed or incomplete immunity, enabling PEDV to persist rather than be eliminated and facilitating its transition from an epidemic pathogen to an endemic component of global swine production systems [3, 6].

A central obstacle to PEDV control, and a defining feature of its relevance as a coronavirus model, is the disconnect between systemic immune responses and effective mucosal protection. Protection of neonatal piglets depends primarily on lactogenic immunity mediated by secretory IgA antibodies delivered through colostrum and milk [3, 11]. Although systemic vaccination can induce strong serum antibody responses in sows, it frequently fails to generate sufficient mucosal immunity, allowing continued viral transmission and recurrent outbreaks [3, 11, 26]. This limitation reflects a broader challenge in controlling enteric coronaviruses, including PEDV, using predominantly parenteral vaccination strategies.

Beyond viral genetics and host immunity, system-level factors also play a critical role in PEDV persistence. These include viral stability in cold environments, prolonged survival in feed and feed ingredients, animal movement patterns, and the high connectivity of modern transport and production networks [6, 27–29]. Epidemiologic and network-based studies consistently identify farm connectivity and animal movement as major predictors of outbreak risk, emphasizing that PEDV persistence is driven not only by viral biology but also by the structural organization of contemporary livestock production systems [28, 29].

In this review, we integrate historical, molecular, immunologic, and epidemiologic evidence to demonstrate that PEDV is not only a long-standing swine pathogen but also a powerful model for understanding how coronaviruses evolve and persist under immune pressure in intensively managed animal populations. By examining PEDV through One Health and systems-resilience frameworks, we aim to identify principles that can inform sustainable coronavirus management strategies in livestock and provide insights applicable to coronavirus biology beyond swine production systems [3, 6].

## Historical timeline and global spread (1971–2025)

### Early European emergence (1971–1980 s)

1971 — Porcine epidemic diarrhea (PED) was first recognized as a distinct clinical syndrome in swine herds in England. The disease was characterized by severe watery diarrhea, vomiting, dehydration, and high mortality in suckling piglets. At the time, the causative agent was unknown, and early cases were frequently misdiagnosed as transmissible gastroenteritis (TGE). In 1972, Oldham (28) described widespread outbreaks among growing and fattening pigs across the United Kingdom and noted how rapidly the infection spread between herds, even when strict disinfection practices were in place. This work highlighted key epidemiological features, e.g., short incubation time, fecal-oral transmission, and herd-level morbidity. This raised the possibility of a novel enteric pathogen distinct from TGEV, thereby prompting further etiological investigations into coronavirus-like particles, based on epidemiological features [30].

1978 — A novel coronavirus was isolated from diarrheic pigs in Belgium, confirming the etiologic agent of PED. This seminal work by Pensaert and De Bouck (1978) represented the first successful isolation and characterization of the virus, establishing it as a distinct enteropathogenic coronavirus unrelated to TGEV [7]. The prototype strain, CV777, became the foundation for subsequent experimental infections, diagnostic assay development, and vaccine design. Shortly after its discovery, Hofmann and Wyler (1988) propagated CV777 in Vero cells and demonstrated that the virus induced severe villous atrophy and enterocyte loss in experimentally infected pigs [8]. These early studies confirmed the fecal-oral route of transmission, tropism of the virus for small intestinal enterocytes, and the characteristic histopathologic lesions that define infection with the newly discovered PEDV. The isolation of the Belgian strain of PEDV, followed by early pathogenesis studies, laid the groundwork for subsequent research and diagnostic development, and ultimately enabled later advances in molecular and epidemiologic characteristics of the virus [7, 8, 31].

### Expansion across Asia (1980–1990 s)

1982–1992 — The PEDV spread to Asia in the early 1980s, with the first major outbreaks reported in Japan, marking the beginning of the virus’s long-term establishment in East and Southeast Asia. Early studies by Takahashi et al. (1983) provided virologic confirmation of PEDV in Japanese herds suffering from severe diarrhea and high mortality among suckling piglets. They demonstrated that the virus had crossed continents and adapted rapidly to intensive swine production systems [32]. Additional outbreaks occurred between 1982 and 1984 in several Japanese prefectures. Microscopic and immunofluorescent examinations revealed damage to the small-intestinal villi that was almost identical to that observed in European herds. Within a short period, antibody surveys showed widespread infection among Japanese farms, even where clinical signs were mild or absent [32]. By the mid-1980s, cases were also appearing in South Korea and Taiwan, particularly during the winter months when hygiene and temperature control were most difficult [33, 34]. Laboratory tests confirmed that PEDV differed from TGEV. Early attempts to control the disease with TGEV-based vaccines were unsuccessful [33, 35].

Through the late 1980s and early 1990s, PEDV became established across much of East Asia. The ability to culture the virus in Vero cells in 1992 made it possible to study replication and immune response under controlled conditions [9]. Field observations summarized by Shibata et al. [10] later showed that outbreaks continued to recur seasonally, particularly during winter months, and that severe disease persisted in neonatal piglets born to sows lacking protective immunity. The disease appeared even in herds with improved sanitation and biosecurity measures [10]. By the early 1990s, PEDV was recognized as one of the main causes of neonatal diarrhea and death in piglets in Japan, South Korea, Taiwan, and Thailand, setting the stage for the severe regional epidemics that followed in the mid-1990s [10, 35].

1993–1996 — Between 1993 and 1996, large-scale PEDV epidemics swept through several Asian countries, particularly Japan, South Korea, and Taiwan. In Japan, severe outbreaks began in late 1993 and persisted through early 1994, causing extensive losses among suckling piglets, with herd-level mortality ranging from 30% to 100% in suckling pigs [33]. The situation intensified in 1996, when a widespread epidemic involving numerous farms across several Japanese prefectures caused substantial mortality in neonatal piglets [33].

Molecular and epidemiologic investigations during this period showed that circulating PEDV strains were highly virulent, well adapted to intensive farrow-to-finish production systems, and capable of rapid farm-to-farm transmission, often via contaminated fomites and personnel movement [34, 36]. In South Korea, PEDV was officially identified in 1992, although later antibody surveys indicated that the virus was likely present since 1987. By the mid-1990s, the virus had become established in many regions, causing repeated seasonal outbreaks [33]. Taiwan and Thailand faced similar problems during this time, reporting wintertime waves of diarrhea in piglets with high fatality rates, even on farms that had adopted rigorous cleaning and disinfection routines [33, 37].

These severe losses revealed just how easily PEDV could spread and survive in farm environments. What had once been an occasional infection was now a constant regional threat throughout East and Southeast Asia. The continuing outbreaks also prompted renewed interest in disease control, leading to the first PEDV-specific vaccine developed in Korea in the mid-1990s [33, 35].

1997–2000 — After the severe epizootics of the early 1990s, PEDV activity declined in Japan and South Korea, entering a phase of lower apparent prevalence but continued enzootic circulation. Diagnostic records from South Korea showed that PEDV remained widespread during this period. From August 1997 to July 1999, examination of 1,258 intestinal samples collected from pigs in five provinces identified 634 cases (50.4%) as PEDV-positive, demonstrating that the virus continued to circulate actively even though large-scale epidemics had subsided [13]. During this time, partial spike (S) gene sequencing of Korean field isolates revealed early diversification into several phylogenetic clusters and substitutions within neutralizing epitope regions, indicating the start of antigenic drift from classical CV777-like strains [13, 33].

Across East and Southeast Asia, sporadic winter outbreaks persisted, often associated with biosecurity lapses, contaminated fomites, or cold-season housing [33]. In Taiwan, the virus remained endemic, producing small-scale winter recurrences almost annually. Serologic and field observations suggested that partial herd immunity in previously exposed herds mitigated mortality but failed to prevent infection [33]. Molecular studies on archived isolates from the late 1990s laid the groundwork for tracking future genogroup transitions that became more apparent in the 2000s [13, 33].

### Transition to highly virulent epidemics (2000–2012)

2000–2007 — Throughout the early 2000s, PEDV persisted across Asia at low to moderate levels, but its genetic and antigenic profiles were quietly changing. Molecular surveillance studies from this period revealed that circulating strains were diverging progressively from the classical CV777 prototype through point mutations and recombination events within the spike (S) gene, especially in regions encoding neutralizing epitopes [13, 33]. This gradual antigenic drift resulted in the emergence of genotypes with distinct phylogenetic signatures, later classified as Group 1 (classical) and Group 2 (variant) lineages [13, 33].

In China, after nearly a decade of sporadic detections, large-scale outbreaks re-emerged in 2006, spreading rapidly through major swine-producing provinces such as Guangdong, Jiangsu, and Henan, with high mortality in suckling piglets [38]. Genetic sequencing of these Chinese isolates showed several amino acid changes in the spike (S) gene compared with the classical CV777 strain. Phylogenetic analyses indicated that the viruses circulating at that time were not direct descendants of older vaccine strains but appeared to have evolved locally or through recombination events within regional herds [38, 39].

A similar pattern was documented in Thailand and Vietnam, where field cases between 2004 and 2007 showed severe villous atrophy and piglet mortality rates approaching 80%, markedly higher than during the preceding decade [37]. These renewed epidemics coincided with the widespread deployment of live and inactivated vaccines developed from outdated, antigenically mismatched strains, an intervention that may have offered partial protection while inadvertently exerting immune selection pressure and facilitating viral adaptation [11, 33]. A combination of genetic and field data from this period shows that PEDV was gradually evolving. The early 2000s were a transition phase, bridging the relatively stable strains of the 1990s and the more aggressive, genetically diverse forms that started to appear after 2008 [11, 13, 33].

2008–2010 — Across East and Southeast Asia, PEDV began showing marked genetic change. In Thailand, severe outbreaks that started in late 2007 were confirmed to be caused by Chinese-like strains; phylogenetics placed the Thai viruses in the same clade as China’s JS-2004-2, signaling regional movement of G2 epidemic lineages [37]. In China, after several years of sporadic detections, a large nationwide wave began around late 2010, with very high mortality in suckling piglets and rapid spread across major swine-producing provinces, evidence that new, more virulent variants were being established [38, 39].

Original molecular work from China showed that 2006–2008 field isolates already differed substantially from the classical CV777 vaccine strain. Full and partial genome analyses documented multiple amino-acid substitutions in the spike (S) gene and phylogenetic separation from vaccine-derived lineages, consistent with local evolution and recombination [38, 40]. Complementing this, Korea deposited a 2008 field variant with a large deletion in the S gene (MF3809/2008), highlighting structural changes in the spike glycoprotein that foreshadowed later S-indel patterns seen in diverse G2 sublineages [41]. Evidence accumulated during this period indicates that recombinant PEDV strains were circulating in Asia, aligning with the surge of highly virulent outbreaks that followed.

### North American introduction (2013–2014)

2013— In early 2013, PEDV was introduced into the United States, marking the first detection of the virus in North America and triggering one of the most severe epizootics in the U.S. swine population. The first cases were reported in April 2013 in Iowa and rapidly expanded to multiple Midwestern states within weeks. Clinical disease was characterized by explosive diarrhea, vomiting, dehydration, and extremely high mortality in suckling piglets, often approaching 90–100% in naïve herds. Molecular characterization of early U.S. isolates revealed that these viruses were highly virulent and genetically distinct from historical European CV777-like strains, instead showing close similarity to contemporary Asian epidemic lineages [19].

Full-genome sequencing of early U.S. strains, including USA/Colorado/2013, demonstrated that the virus belonged to the highly virulent G2 genotype and shared high nucleotide identity with PEDV strains circulating in China during the late 2000s and early 2010s, strongly suggesting recent transcontinental introduction [19, 21]. Within months of its detection, PEDV spread to more than 30 U.S. states, facilitated by the high connectivity of modern swine production systems, extensive animal movement, and contaminated transport vehicles. By the end of 2013, PEDV had become endemic in many major swine-producing regions of the United States, resulting in the loss of millions of piglets and substantial economic damage to the pork industry [18, 21].

2014–2015 — In 2014, alongside the highly virulent “original” U.S. PEDV strain, a genetically distinct S-INDEL variant (with characteristic small insertions and deletions in the spike gene) was identified in North America. The prototype S-INDEL strain OH851 was first described in Ohio, where full-genome sequencing showed three deletions, one insertion, and multiple point mutations in the S1 region compared with earlier U.S. epidemic strains [20]. Subsequent surveillance demonstrated that prototype (non–S-INDEL) and S-INDEL PEDV strains were co-circulating in multiple U.S. states between 2013 and early 2014, with the S-INDEL viruses forming a distinct phylogenetic cluster within the North American clade [21].

Experimental infection studies with a U.S. S-INDEL strain (Iowa106) in conventional nursing piglets showed that, although morbidity remained close to 100%, mortality, intestinal lesions, and the duration of diarrhea were significantly lower than those induced by the original highly virulent U.S. strain PC21A. Prior exposure to the S-INDEL virus also conferred partial cross-protection against challenge with the prototype strain, supporting the concept that these S-INDEL lineages generally represent milder but still clinically relevant variants [12].

At the same time, PED re-emerged in Europe. In 2014, acute outbreaks were reported on pig farms in southern Germany, where sequencing revealed that the causative viruses were clearly different from historical European CV777-like strains and instead clustered closely with U.S. S-INDEL PEDV variants [42, 43]. Follow-up molecular work from Italy documented that PEDV detected in Germany, France, and Belgium during 2014–2015 had high nucleotide identity to U.S. S-INDEL strains, indicating recent transcontinental introduction and subsequent spread within Europe [43].

Together, these findings show that by 2014–2015, classical highly virulent PEDV strains and milder S-INDEL variants were circulating simultaneously across North America and Europe, underscoring the ease of international spread and the growing challenge that antigenic and genetic diversity poses for effective vaccine protection [12, 20, 21, 42, 43].

### Global emergence of large spike-deletion variants (2014–2017)

2016–2017 — During this period, PEDV evolution was marked by the emergence of large spike-gene deletion variants, often detected together with classical highly virulent strains. In Japan, Diep et al. [22] identified multiple PEDV genotypes carrying 582–648 nt deletions in the S1 N-terminal region, many of which were found alongside intact S-gene PEDV within the same herd, indicating ongoing mixed infections and rapid viral diversification under field conditions [22]. Earlier reports had already described the Tottori2 strain, a Japanese variant possessing a 582-nt S-gene deletion, showing that strains with such deletions had been circulating for several years [23].

Similar findings emerged in North America. In the United States, surveillance between 2016 and 2017 detected S1 N-terminal deletion variants with 194- or 204-aa deletions that were repeatedly identified together with the original U.S. epidemic strain, confirming active co-circulation of divergent PEDV lineages [24]. Experimental studies demonstrated that a related 197-aa S1 deletion produced an attenuated clinical phenotype in piglets, while also reducing the breadth of protective immunity, a combination that may complicate vaccine matching and immune control [25]. Large-deletion PEDV also appeared in South Korea, where a variant with a substantial genomic deletion was reported as early as 2014, illustrating that independent emergence of major S-gene deletions had occurred in multiple countries across Asia [41]. Taken together, these findings show that by 2016–2017, large S-deletion PEDV variants were coexisting and co-infecting pigs alongside classical highly virulent strains in Japan and the U.S., with similar variants documented in South Korea. Obviously, this persistent diversification of the spike gene under field conditions has important implications for the design of diagnostic assays and selection of vaccine strains.

### Genetic diversification and global endemicity (2018–2025)

2018–2019 — By 2018, PEDV had moved into a sustained endemic phase across major swine-producing regions, while new data helped clarify how the virus persisted and spread. In Europe, a detailed investigation of the 2015–2017 wave in Italy documented hundreds of PEDV-positive farms, with most clustered along major transport networks. Molecular sequencing showed that these European viruses were closely related to U.S. S-INDEL–like lineages, and network analysis identified pig and truck movements as key drivers of regional transmission [43]. At the same time, in North America, sequence-based surveillance confirmed the co-circulation of highly virulent U.S. G2b strains and newly emerging S1 N-terminal deletion variants, which carried a ~ 200 amino acid deletion in the S1 domain. Su et al. [24] identified several such deletion variants in U.S. herds, often detected together with classical strains in diagnostic samples, indicating ongoing mixed infections and active viral diversification.

By 2019, research focus shifted to understanding why PEDV continued to spread despite heightened awareness and biosecurity. A large-scale U.S. study analyzing 1,897 farms and weekly data from 332 sow herds used machine-learning models to show that incoming pig movements within a 10-km radius were the strongest predictor of PEDV outbreaks, followed by local hog density and environmental features such as temperature and vegetation. The model predicted weekly outbreak status with > 80% accuracy, underscoring the combined influence of animal movement and environmental suitability in endemic PEDV transmission [28].

At the same time, epidemiologic work in China confirmed that PEDV remained widespread. A 2019 systematic review and meta-analysis of Chinese data estimated a high pooled prevalence of PEDV infection across pig herds, with significant regional variation but persistent circulation in most major production areas [44]. Genomic surveillance also continued; the complete genome sequence of a variant strain ZJ/ZX2018-C10, isolated from piglets with diarrhea in Zhejiang province, revealed additional mutations and insertions in the spike gene on a G2b backbone, illustrating ongoing diversification of epidemic lineages [45].

Together, these studies showed that even several years after the initial global epidemics, PEDV remained genetically dynamic and epidemiologically entrenched, with transmission driven by complex interactions between virus evolution, animal movement, and farm-level biosecurity. In addition, genetic diversification continued through both S-gene alterations and geographically linked lineage turnover [6, 44, 45].

2020–2022 — During this period, large-scale molecular surveillance confirmed that PEDV remained genetically diverse but was dominated by variant G2 lineages, especially G2b. In China, a nationwide study analyzing 91 full-length spike (S) gene sequences from PEDV-positive farms in 17 provinces (March 2020–March 2021) showed that 92.3% of strains belonged to genotype GII (mostly GII-b), while only 7.7% were S-INDEL–like strains grouped within the GI-c clade [46]. Recombination analysis demonstrated that several GI-c S-INDEL–like viruses were recombinants between the European S-INDEL strain FR/001/2014 and the highly virulent Chinese AJ1102 strain. Multiple novel insertions (e.g., 360QGRKS364 and 1278–1281VDVF) and amino-acid substitutions were identified within key neutralizing epitopes of S compared with CV777- and AJ1102-based vaccines, indicating ongoing antigenic drift and vaccine mismatch [46].

A provincial survey from Shandong Province used samples collected between 2019 and 2021. This survey found that all sequenced isolates belonged to the G2 genotype, again confirming that variant G2 strains had replaced classical G1 viruses as the dominant field strains in this important swine production region [47]. In Europe, a molecular study from Poland using 662 fecal samples collected from 2015 to 2021 showed that PEDV was present at low prevalence (3.8%) but that circulating strains belonged to the G1b (S-INDEL) subgroup and carried a ~ 400-nt recombinant fragment at the 5′ end of the S gene derived from swine enteric coronavirus (SeCoV), demonstrating that recombinant PEDV/SeCoV-like viruses were persisting in European herds during this timeframe [48].

In the United States, PEDV circulated at relatively low, endemic levels during 2020–2022. Diagnostic submissions still identified both the original U.S. G2b strains and the previously described S1–N-terminal deletion variants, but no large, nationwide outbreaks were reported [29, 49]. Experimental and genomic work during this period also showed that changes outside the spike gene may influence how PEDV behaves. One newly identified strain carried a deletion in the N gene. Infection studies in piglets demonstrated shifts in both disease severity and immune response, indicating that non-spike genes can also affect virulence and vaccine effectiveness [50]. Another study described a field strain with a naturally truncated ORF3 gene co-circulating with full-length ORF3 viruses. Experimental infection in piglets indicated that ORF3 deletions influenced viral replication dynamics and that they may serve as genetic markers for attenuation or as vaccine candidates [16].

Taken together, surveillance data from 2020 to 2022 show that PEDV remained stratified into multiple lineages, with G2b and related variant genotypes dominant and S-INDEL/G1b strains persisting at lower levels, while frequent recombination and mutation in the S, N, and ORF3 genes continued to shape transmission, tissue tropism, and immune escape [16, 46–48, 50]. This historical trajectory from a localized enteric disease described in the 1970s to a globally distributed epizootic virus supports the view that PEDV is a long-term One Health concern within animal–environment systems, requiring integrated surveillance and control strategies (including updated vaccines and biosecurity programs) that explicitly account for ongoing viral evolution at the animal–environment interface [3].

2023 — The global situation in 2023 was mixed. In some places PEDV persisted quietly at endemic levels, but in others it reappeared more forcefully, largely due to the emergence of recombinant variants. Genomic surveillance from China, using samples collected during 2022–2023, identified several recombinant G2b lineages. These viruses contained mosaic spike regions formed from both classical G1 and virulent G2 donors. The resulting strains showed notable amino-acid changes in the S1 NTD and COE neutralizing domains, raising ongoing concerns about antigenic drift and the fit between circulating viruses and existing vaccines [51]. Several provinces also reported small clusters of severe diarrhea in young piglets, and sequence data linked these episodes to newly emerging G2b–G1 recombinant strains [51].

In Southeast Asia, particularly in Vietnam, diagnostic laboratories reported increased PEDV detections compared with the previous two years. National surveillance data indicated that G2b viruses and S-INDEL–type G1 strains continued to circulate, sometimes appearing together in the same outbreaks, a pattern similar to what had been documented in Korea and Japan [52].

In North America, PEDV remained endemic but largely restricted to sporadic breakouts in sow farms. Updated U.S. monitoring data showed intermittent detection of PEDV in states such as Iowa, Minnesota, and Oklahoma, though at markedly lower levels than pre-2018 patterns [29, 49]. Viral genomes recovered from these cases showed continued circulation of the established U.S. G2b lineage, with no evidence of the emergence of new dominant genotypes, suggesting that ongoing low-level transmission was driven primarily by herd-to-herd spread rather than viral evolution [53].

2024 — By 2024, genomic studies demonstrated that the global PEDV population remained dominated by G2b lineages, but recombination continued to contribute to genetic diversity. A large-scale evolutionary analysis of PEDV in the United States, a decade after its initial 2013 introduction, revealed ongoing lineage turnover within G2b. Multiple co-circulating clusters are showing modest accumulation of amino-acid substitutions in the S1 domain [53]. Experimental work also showed that antigenic changes in circulating strains can reduce the protective match with existing vaccines. In 2020, Wang et al. [50] demonstrated that deletions and sequence variation in structural genes, including regions outside the spike protein, can alter immunogenicity and affect the degree of protection provided by the current vaccine strains.

In East Asia, Chinese and Korean researchers reported the detection of additional recombinant PEDV genomes containing fragments derived from SeCoV, confirming that interspecies recombination continued to shape PEDV evolution [5, 48]. These recombinant viruses maintained the overall structure of the virulent G2b lineage, but changes in the S1 region, including insertions and deletions, modified key antigenic sites. Such alterations have made diagnostic interpretation more difficult and have created uncertainty about how these strains behave in infected herds [5]. Late in 2024, reports from China and Thailand again mentioned PEDV activity during the winter months, a timing that has been seen repeatedly in earlier years. The most serious losses occurred in herds where sows did not provide adequate lactogenic immunity, and these cases showed moderate to high piglet mortality, reinforcing the need to improve maternal vaccination programs [26].

2025 — Recent research underscores that PEDV remains an endemic, evolving pathogen with sustained epidemiologic significance. A 2025 analysis of PEDV in U.S. herds demonstrated that low-level circulation persisted with no major new introductions, but the virus continued to undergo slow but steady evolution, particularly in the S1 region and accessory proteins [49]. This study confirmed that the U.S. PEDV strains were derived from the original 2013 introduction but still exhibited new mutations affecting antigenic regions relevant to vaccine escape [49].

Detection of PEDV increased in many countries during 2025; some of these events were linked to contaminated feed ingredients or the movement of pigs and pig products over long distances, routes that were also implicated during the 2013–2014 outbreaks in North America [27]. Concurrently, researchers documented nonrandom recombination among swine enteric coronaviruses, including PEDV, which contributed to the emergence of mosaic genomic structures and raised new questions about cross-species interactions under a One Health framework [5]. These findings highlight the ongoing relevance of environmental reservoirs, feed-related risks, and interspecies viral interactions in shaping the future trajectory of PEDV outbreaks.

It has now become clear that PEDV is not going away. The virus keeps circulating via a combination of gradual genetic changes, animal movements, and gaps in mucosal immunity in breeding herds [54]. Continued genomic surveillance, updated vaccine matching, and international coordination remain essential to reduce future disease burden.

## Genomic organization and viral structure

### Genome architecture (~ 28 kb)

PEDV is an enveloped, pleomorphic, single-stranded RNA virus classified within the genus *Alphacoronavirus*. The virions are generally spherical or slightly ovoid and usually measure around 100–150 nm in diameter, although their size and shape can vary depending on the host cell in which they were produced. In some in vitro cultures, longer or somewhat irregular particles are also seen. The lipid bilayer envelope is densely ornamented with the large trimeric spike (S) glycoproteins, which protrude prominently from the surface and give the virus its characteristic corona-like appearance. Embedded between these spikes are fewer copies of the envelope (E) and membrane (M) proteins, which coordinate virion assembly, curvature, and budding. The structural proteins are anchored into the host-derived membrane, beneath which lies the ribonucleoprotein (RNP) core composed of genomic RNA wrapped within the nucleocapsid (N) protein [3, 11].

The PEDV genome is a single-stranded, positive-sense RNA molecule approximately 28 kilobases in length, making it one of the largest known positive-sense RNA viral genomes. It carries the canonical coronavirus gene order: 5′-UTR–ORF1a/1b–S–ORF3–E–M–N–3′-UTR. Roughly two-thirds of the genome comprises ORF1a and ORF1b, which encode two large replicase polyproteins, pp1a and pp1ab. These polyproteins are processed into a suite of nonstructural proteins (nsps) that collectively form the replication–transcription complex responsible for viral RNA synthesis, proofreading, and the generation of sub-genomic RNAs. The remaining one-third of the genome encodes the major structural proteins—S, E, M, and N—along with the accessory gene ORF3, which is unique to PEDV. It plays a notable role in modulating viral fitness, cell survival, and virulence [3, 11, 14, 50].

### Spike (S) glycoprotein

The spike (S) glycoprotein is the most functionally and antigenically important structural component of PEDV. It is a large type I membrane protein that is post-translationally cleaved into S1 and S2 subunits. S1 subunit, which contains the receptor-binding domain, harbors several major neutralizing epitopes, including COE, SS2, and SS6, all of which are critical targets for protective immunity. The S2 subunit mediates membrane fusion and contains the heptad repeat regions essential for viral entry. Variation within the S gene is responsible for much of the phenotypic diversity observed among PEDV strains. A key distinction is between non–S-INDEL variants, which possess intact S1 domains and are responsible for severe, high-mortality outbreaks such as those observed in the United States in 2013–2014, and S-INDEL strains, which contain characteristic insertions and deletions within the S1 N-terminal region and typically produce milder disease [21, 33]. Over the past decade, multiple countries have reported the emergence of large S-gene deletion variants, with deletions ranging from 194 to over 600 nucleotides, affecting the N-terminal half of the S1 region. It is important to realize that, despite their generally lower virulence, these deletion-type strains may stimulate only partial immunity, which leads to the continued co-circulation of both attenuated and fully virulent viruses within the same geographic area [3, 11, 22, 24].

### ORF3 accessory protein

ORF3 is located between the S and E genes and encodes the major accessory protein of PEDV. This genomic region shows significant sequence variation among circulating strains. Experimental studies show that ORF3 interferes with apoptotic pathways, allowing infected epithelial cells to survive longer and increasing viral replication. The protein is also thought to contribute to ion channel activity and intracellular trafficking, although several aspects of its function remain incompletely defined. Importantly, naturally occurring truncations of ORF3, commonly seen in cell-culture–adapted viruses and occasionally detected in field strains, are closely linked with attenuated phenotypes. For this reason, ORF3 deletions have become a practical molecular marker to distinguish attenuated strains from their wild-type counterparts [3, 11, 14, 15].

### Membrane (M), Envelope (E), and Nucleocapsid (N) Proteins

The membrane (M) protein is the most abundant structural component of the PEDV virion. It plays a key role in assembling new virions by interacting with the E, S, and N proteins to drive envelope formation and maintain the virus’s structural integrity. Although the envelope (E) protein is quite small, it participates in several critical stages of the viral life cycle, including membrane scission, virion budding, and the generation of ion channels within infected cells. Despite its modest size, the E protein is required for efficient virion production [55, 56].

The nucleocapsid (N) protein binds the viral genomic RNA to generate the characteristic helical ribonucleoprotein complex. In addition to its role in RNA packaging, N interacts with components of the replication–transcription machinery to improve RNA synthesis and modulates host innate immune responses. Recently identified N-gene deletion variants appear to affect both replication dynamics and virulence, underscoring the importance of this protein in PEDV biology [3, 11, 15, 33].

The combined activities of the S, M, E, and N structural proteins, and the regulatory influence of ORF3, enable efficient viral entry, replication, assembly, and dissemination within the porcine small intestine. The marked variability in the S and ORF3 regions, together with the large size of the coronavirus genome, allows PEDV to adapt genetically with ease. This ongoing capacity for change promotes the appearance of new variants and continues to challenge long-term control efforts [3, 11].

## Pathogenesis, tissue tropism, and immune response

The way PEDV causes disease is closely linked to how the virus replicates, the biology of the intestinal lining, and the immune limitations of newborn piglets. This virus mainly infects the cells that absorb nutrients in the small intestine. The disease severity is dependent on the virus virulence, age of the pig, the rate of intestinal lining regeneration, and the level of immune protection in the gut [10, 33, 57]. Infected animals usually develop sudden watery diarrhea, vomiting, dehydration, and, if they do not have enough protection from their mother’s milk, very high death rates in newborn piglets [3, 33, 57].

After swine are exposed to the virus orally, they shed large amounts of the virus in their feces. This makes it easy for the virus to spread through fecal-oral transmission within farrowing units and between groups of pigs [33, 57]. The virus multiplies quickly, and the damage and viral proteins are mostly found in the small intestine [3, 57]. This injury to the intestinal lining interferes with absorption and weakens the barrier, leading to the severe fluid loss seen in serious cases of PED [3, 33, 57].

### Intestinal tropism

The PEDV mainly targets mature villous enterocytes in the small intestine, especially in the jejunum and ileum, following both natural and experimental infections [2, 3, 10, 33]. The virus enters cells using its spike glycoprotein, which controls attachment and fusion, and is the main factor for neutralizing antibody recognition and antigenic variation [2, 3, 33]. Since the replication of PEDV depends on the differentiation state of enterocytes, the virus infects mature absorptive cells at the villus tip rather than the crypt progenitors, leading to lesions that are mostly found in the villi [3, 10, 33].

This biology explains why clinical outcomes depend so much on age. Newborn piglets are much more vulnerable because they have a limited ability to regenerate their intestinal lining and less physiological reserve. In addition, they depend mostly on passive mucosal immunity for protection [10, 33]. Older swine usually have lower death rates and recover faster, since their intestinal lining renews more quickly and their immune systems are more developed, although they can still get infected and shed the virus [3, 33]. PEDV is mainly known as an enteric coronavirus, and both field and experimental studies show that it mostly replicates and causes damage in the small intestinal mucosa. This highlights the importance of strong protective immunity at the gut surface to prevent severe disease [2, 3, 33].

### Mechanisms of injury

Intestinal injury in PEDV infection occurs primarily through the direct loss of mature absorptive enterocytes and the subsequent downstream physiological consequences of villous collapse. Viral replication in villous enterocytes leads to cellular dysfunction and death, resulting in villous atrophy, a reduction in the absorptive surface area, impaired digestion and transport, and the rapid development of malabsorptive/osmotic diarrhea [3, 33, 57]. The severity of dehydration reflects both reduced absorptive capacity and disruption of epithelial barrier function as enterocytes slough from villus tips, amplifying fluid and electrolyte loss, effects that are most lethal in neonatal piglets [10, 33, 57].

Innate immune responses shape both restriction of viral replication and the inflammatory environment within the gut. PEDV triggers mucosal antiviral signaling, including interferon-linked pathways; however, PEDV also encodes functions that antagonize or reshape host responses, contributing to efficient replication and continued shedding [3, 33]. A mechanistic example is the PEDV accessory protein ORF3, which has been shown experimentally to enhance viral proliferation by inhibiting apoptosis of infected cells, thereby extending the productive lifespan of infected enterocytes and increasing viral output [14]. Such virus–host interactions can intensify epithelial injury indirectly by sustaining infection in the very cell population that maintains fluid and nutrient absorption.

Adaptive immunity is critical for limiting reinfection and reducing clinical severity in older animals. However, it is often too slow to prevent acute injury during primary infection in neonates. As a result, the best way to protect newborns is through lactogenic immunity, especially secretory IgA from colostrum and milk, which neutralizes the virus at the gut lining and helps prevent damage to the villi [33, 57]. Thus, humoral antibodies from injected vaccines may not always protect piglets but might lower the death rates and still allow viral infection and spread [33, 57].

Together, these mechanisms position PED as a disease due to the high-efficiency viral replication within a developmentally constrained epithelial niche, amplified by the physiological fragility of early life and constrained by the requirement for mucosal (rather than purely systemic) immune protection [3, 10, 33, 57].

### Lactogenic immunity and the gut–Mammary–secretory IgA axis in PEDV protection

Protection of neonatal piglets against PEDV depends predominantly on passive lactogenic immunity rather than on the piglet’s endogenous immune response or maternal serum antibody levels alone. Studies have shown that secretory IgA (sIgA) in colostrum and milk is the main factor protecting suckling piglets from severe diarrhea, gut damage, and death. This immunologic requirement reflects the strict enteric tropism of PEDV and the limited capacity of neonatal piglets to mount effective adaptive immune responses in early life. Comprehensive reviews of PEDV immunoprophylaxis consistently emphasize that maternal milk–derived antibodies, particularly IgA, are essential for neutralizing the virus at the intestinal mucosal surface where viral replication occurs [33, 57].

Lactogenic immunity is based on the gut–mammary immune axis, a pathway found in swine and other mammals. When swine are exposed to PEDV, either naturally or through immunization, antigen-presenting cells in the gut take up the virus and activate B cells in Peyer’s patches and nearby lymph nodes. These B cells become IgA-secreting cell precursors, which develop receptors that help them move to the gut and mammary gland. They then travel through the body to the mammary gland during late pregnancy and nursing. Once localized within mammary tissue, these ASCs secrete PEDV-specific sIgA into milk, thereby providing continuous mucosal protection to nursing piglets. This mechanism has been conclusively demonstrated in controlled challenge studies showing that oral exposure of pregnant gilts to live PEDV induces robust milk IgA responses and confers complete protection to suckling piglets against virulent PEDV [10, 33].

The timing of maternal antigen exposure strongly influences the development of lactogenic immunity through the gut–mammary axis. When sows encounter antigen earlier in gestation, more robust induction and trafficking of IgA-secreting cells to the mammary gland are reported. By contrast, exposure near the farrowing tends to produce lower milk IgA levels and less consistent protection in nursing piglets. Taken together, these observations indicate that gestational stage shapes the strength and reliability of lactogenic protection, with direct implications for vaccine scheduling and controlled exposure strategies [33].

Maternal serum IgG levels do not reliably predict protection of newborn piglets, underscoring a key limitation of parenteral vaccines for enteric coronaviruses. Systemic immunization can raise blood IgG levels but often fails to provide sufficient intestinal priming or gut-homing IgA ASCs, leading to weak lactogenic immunity. Studies have shown that milk IgA levels and intestinal neutralizing activity are more closely linked to lower viral shedding and better clinical outcomes than serum IgG alone [33]. This explains why sow herds with strong humoral antibody levels can still have severe PEDV outbreaks if mucosal immunity is lacking.

Recent studies have shown that PEDV is a useful model for understanding mucosal immunity against enteric coronaviruses, which is important for vaccine development. Long-lasting protection against PEDV depends on vaccines or exposure methods that stimulate the gut and mammary immune systems, rather than on the general immune response [3, 33, 54]. Overall, these findings show that lactogenic immunity is key in controlling PEDV in breeding herds and highlight the importance of vaccine approaches and management practices that focus on priming intestinal immunity and maintaining milk sIgA levels.

### Why systemic vaccination often fails

Although commercial vaccines are commonly used, systemic immunization does not protect newborn piglets against PEDV. This is due to the basic principles of mucosal immunology, rather than problems with the vaccines. PEDV replication is largely restricted to mature enterocytes of the small intestine, and hence effective protection requires immune effectors capable of neutralizing the virus at the intestinal mucosal surface. Systemic vaccination reliably induces circulating IgG responses but often fails to provide sufficient intestinal immune priming or to activate the gut–mammary immune axis, resulting in inadequate delivery of protective secretory IgA (sIgA) to nursing piglets [33, 57].

Studies comparing different vaccination methods show that high maternal serum IgG titers do not correlate with protection of suckling piglets when mucosal immunity is weak. On the other hand, higher levels of sIgA in milk are linked to less viral shedding, less gut damage, and better survival in piglets [10, 33, 57]. Oral or mucosal administration of vaccines is more effective at inducing gut-targeted IgA-producing cells than intramuscular or subcutaneous injection, which primarily stimulates systemic immune tissues rather than gut tissues. This distinction accounts for the observation that injected vaccines may demonstrate efficacy in laboratory assays but exhibit reduced effectiveness in real-world conditions.

Field observations support these experimental results. Sow herds that receive parenteral vaccines often develop strong antibody levels, but still face severe PEDV outbreaks in newborn piglets, especially if vaccination happens late in pregnancy or during an outbreak. Emergency vaccination during outbreaks can trigger immune responses in sows, but often does not provide enough lactogenic protection in time, since there is not enough time for intestinal priming, IgA ASC expansion, and movement to the mammary gland before birth. These issues are well documented in controlled studies and help explain why inactivated and attenuated injectable PEDV vaccines show inconsistent results in different production systems [11, 57].

Recent studies measuring antibody responses have helped explain the gap between systemic immunity and actual protection. Detailed immunoprofiling shows that neutralizing activity in milk and intestinal secretions, not in serum, is the best predictor of protection against PEDV. This highlights that systemic antibodies alone are not enough to control enteric coronaviruses. Analyses of post-infection and post-vaccination immune responses show that although both IgG and IgA antibodies are induced, only mucosal IgA responses correlate strongly with protection against clinical disease and suppression of viral replication in piglets [10, 57]. These findings underscore a central challenge in PEDV immunoprophylaxis: vaccines optimized for systemic immunity may fail to engage the immune pathways required for effective mucosal defense.

The challenges of parenteral vaccination are not limited to PEDV but are seen in other enteric coronaviruses as well. Similar issues have been reported with transmissible gastroenteritis virus (TGEV) and other gut pathogens, showing that this is a broader biological issue, not just a problem with PEDV. These findings suggest that we should move away from relying only on systemic immunization and instead focus on vaccine approaches that boost mucosal immunity, especially during pregnancy when the gut-mammary axis is most active. This understanding has renewed interest in oral, mucosal, vectored, and nucleic-acid–based vaccines to address the limits of parenteral PEDV vaccination [11, 57].

### Next-generation vaccine platforms

Because systemic vaccination has its limits, new PEDV vaccines are being developed to trigger strong mucosal immunity, especially by activating the gut-mammary-secretory IgA pathway. These new methods try to solve the problem that enteric viruses replicate in the gut, while traditional vaccines work through the bloodstream, by delivering antigens directly to mucosal tissues or using platforms that can trigger a strong immune response in the intestines. The most promising strategies include rationally attenuated live vaccines, oral vector systems, and nucleic acid-based platforms, all of which have the potential to provide lasting lactogenic protection [11, 57].

Live-attenuated PEDV vaccines remain the best option for inducing mucosal immunity because they closely mimic natural infection and activate intestinal IgA responses. Studies show that administering live-attenuated PEDV orally results in high milk IgA levels and fully protects suckling piglets from dangerous infections. However, safety issues, such as the risk of the virus becoming harmful again, mixing with other strains, and long-term viral shedding, have limited their use. To reduce these risks, researchers are now using targeted genetic changes, such as deleting parts of the spike (S) gene, shortening the ORF3 accessory protein, and altering nonstructural proteins to make the vaccine more stable while retaining its ability to trigger mucosal immunity [14, 16, 25, 35]. Thus, a careful vaccine design should include a vaccine that is safe and effective.

Oral vectored and probiotic-based vaccine platforms offer another option to deliver PEDV antigens directly to the intestinal mucosa, avoiding the risks associated with replication-competent coronaviruses. Recombinant Lactobacillus, Bacillus, and viral vector systems that express PEDV spike or subunit antigens have been shown to trigger mucosal IgA responses and offer partial protection in experimental models. These platforms are generally safe and easy to produce, but there are still challenges with antigen expression, gut stability, and consistent immune responses in real-world situations. Despite these limitations, oral vector systems are increasingly viewed as valuable tools for herd-level mucosal priming when incorporated into an integrated immunization program [11, 57].

Vaccines based on nucleic acids, such as DNA, mRNA, and circRNA, are increasingly being explored because they enable relatively rapid updates to antigen sequences. These platforms have generally been evaluated for their ability to induce systemic immunity, but recent work indicates that mucosal delivery approaches or combined prime–boost regimens may support the development of intestinal IgA responses. A bivalent circRNA vaccine that expresses PEDV and TGEV antigens has been shown to induce both systemic and mucosal immune responses and to provide partial protection in piglets, highlighting the potential of this platform to control multiple enteric coronaviruses [26, 54]. However, achieving consistent mucosal immunity with nucleic acid vaccines remains technically challenging, and better delivery systems are still being explored [3, 11, 57].

Subunit and spike-based vaccines are also in use to control PEDV, but they have limits. When given by injections, they often do not trigger strong mucosal immunity and can miss the target due to changes in the virus. New adjuvants and ways to deliver these vaccines to mucosal surfaces have helped a little, but they still do not protect as well as live or oral vaccines. Also, the spike gene keeps changing, especially in areas that help the immune system recognize the virus, so subunit vaccines need regular updates based on ongoing genetic monitoring [11, 33, 40].

Overall, these new vaccine approaches highlight an important point for PEDV prevention: strong protection against gut coronaviruses depends on activating mucosal immune responses. No single vaccine type has yet reached the best mix of safety, efficacy, and ease of use. However, improving oral and mucosal vaccines and carefully timing vaccinations in breeding herds seem to be the best ways to achieve lasting PEDV control. Lessons from PEDV vaccine research can also help with other gut viruses and show that mucosal immunity is key to preventing coronavirus infections [11, 54, 57].

### Epidemiology and transmission dynamics

Many factors shape PEDV epidemiology, e.g., viral genetics, how production systems are set up, how long the virus survives in the environment, and patterns of animal and commodity movement. After the 2013–2014 outbreaks in North America, the main routes of virus introduction and spread locally and regionally were found to be through animal movement, contaminated feed, changes in the environment with the seasons, and viral recombination. These factors are now part of how scientists model the disease and plan control strategies [6, 11, 57].

#### Transmission pathways

Fecal–oral transmission: PEDV spreads very efficiently among livestock. Infected swine sheds/releases large amounts of the virus, often reaching 10⁸ to 10¹⁰ genome copies per gram of feces, which helps the virus move quickly through farrowing units and nurseries. Shedding usually begins 24 h before symptoms appear and can last for more than 2 weeks, increasing the risk of virus spread within the herd [10, 33, 57].

Fomite contamination— Contaminated boots, gloves, coveralls, processing tools, needles, farrowing equipment, and pen dividers have all been implicated as mechanical sources of spread. Organic matter significantly reduces disinfectant efficacy. Studies have shown that PEDV can survive on common farm materials—including stainless steel, aluminum, rubber, and plastic- for days to weeks at low temperatures [27, 57].

Transport and marketing networks— Livestock trucks and trailers remain among the main modes of virus spread. In the 2013–2014 epidemic in the United States, large studies showed that contaminated trailers leaving slaughter plants started new outbreaks in different regions. Truck routes connecting these areas made the virus spread quickly. Even after washing, viral RNA was detected in 12–40% of trailers, demonstrating the difficulty of removing all organic material [27, 28].

Animal movement as a major driver of transmission— Several epidemiologic and modeling studies have found that moving animals, especially piglets and replacement gilts, is a strong predictor of PEDV outbreaks. Farms that are close together and move animals often face higher risks, emphasizing how connections between farms and local factors affect outbreaks [6, 37].

#### Feed and ingredient contamination

Prolonged viral survival in feed matrices— Studies show that PEDV can stay infectious for different lengths of time in common feed ingredients like soybean meal, DDGS, lysine HCl, choline chloride, and spray-dried porcine plasma. How long the virus survives depends on temperature, humidity, and the feed ingredient. In some cases, the virus can stay infectious for weeks, even in tests that mimic long-distance transport. These results suggest that contaminated feed ingredients could facilitate the spread of PEDV and highlight the need for robust feed-mill biosecurity and prevention measures [27, 54].

Implications for long-distance spread—PEDV can remain infectious for long periods of time in some feed ingredients, especially when water activity is low, and conditions are similar to long-distance transport. This suggests that contaminated feed ingredients could be a risk factor for PEDV moving across borders, even though it is difficult to directly prove global introduction events. However, field investigations and outbreak reconstructions have found links that support the idea of feed-mediated spread. This highlights the need for strong feed-mill biosecurity, careful ingredient sourcing, and effective mitigation strategies as part of the PEDV control programs [27, 54].

Mitigation strategies—Thermal processing above 70 °C, medium-chain fatty acids, organic acids, and some formaldehyde-based additives have all been shown to be effective at inactivating PEDV in feed. However, their effectiveness depends on the type of ingredient, fat content, and particle size [27].

#### Environmental factors

Climatic influences— PEDV becomes much more stable at low temperatures and low humidity. These conditions slow down viral breakdown and allow the virus to stay infectious longer in manure, slurry pits, transport trailers, loading docks, and on contaminated objects. Studies show that at 4 °C, the virus can remain infectious for at least 28 days, but it decays much faster at 25–37 °C [27].

Seasonality— In multiple regions, PEDV outbreaks tend to occur during colder periods of the year. This pattern is consistent with experimental data indicating that the virus remains more stable at low temperatures and loses infectivity more quickly as temperatures rise. Cold and humid environmental conditions may thus allow PEDV to persist longer in contaminated materials, potentially facilitating transmission during winter months [11, 27, 54].

Environmental reservoirs—The PEDV can survive in contaminated organic material, such as manure, and in farm environments. Its stability in these settings and repeated fecal shedding can lead to ongoing infections and local spread. Once the virus is in the environment, it can be spread via contaminated surfaces, equipment, and objects moving between locations, increasing the risk of indirect transmission within and between production sites [11, 54, 57].

#### Geographic differences

Asia— Asia has been a key region for PEDV genetic diversity, with multiple genotypes, such as G2b and S-INDEL variants, circulating for a long time. Recombinant and mixed viral populations are often found. Ongoing molecular surveillance in countries such as China, South Korea, Thailand, and Japan has shown continued viral evolution and the co-circulation of different PEDV lineages over time [3, 6, 24, 33, 47, 51].

North America— After the major 2013–2014 epidemic, PEDV has continued to circulate in the United States at generally low levels. Most new cases are found in sow herds. Molecular surveillance indicates that genetic changes continue to occur within established lineages, and G2b variants are still being detected regularly. This continued presence suggests that transmission is still happening within and between herds, even though biosecurity practices have improved [20, 21, 49, 53].

Europe— Europe shows a different pattern, with outbreaks happening only occasionally and often involving S-INDEL strains that are less severe. Studies have found cases linked to North American S-INDEL viruses and recombinant PEDV/SeCoV strains. This means Europe is still at risk for new cases through trade or feed imports [42, 43, 48].

#### Epidemiological modeling

Machine-learning and network models— Machine-learning models that use swine density, temperature, elevation, vegetation index, pig movement records, and past outbreak data have reached over 80% accuracy in predicting PEDV outbreaks. Studies show that incoming pig movement is the most important factor, followed by local pig density and environmental suitability [28, 29].

Spatial clustering— Studies that examine where and when outbreaks occur show that PEDV is most often found near transport routes, packing plants, and areas with many swine farms. Special maps show the disease spreads quickly around infected sow farms, especially when the environment is dirty, and pigs are moved often [28, 29].

Model implications— These quantitative models are now used to identify high-risk periods for targeted actions. Examples include better trailer sanitation, feed mitigation, changing transport routes, and regional sow herd vaccination to stop transmission before outbreaks grow [11, 28, 57].

### PEDV-driven economic disruption in modern swine production systems

PEDV is one of the most economically significant enteric pathogens in swine, as it causes a rapid supply shock. This is mainly due to high pre-weaning mortality and short-term drops in pork production, not a decrease in consumer demand. The 2013–2014 North American outbreak showed this clearly, as the main market effects came from fewer swine being available and processing challenges, not from food-safety–related demand loss [18]. When highly virulent PEDV strains entered U.S. breeding herds in 2013, they caused widespread outbreaks, resulting in high piglet mortality and major disruptions to farrowing systems [20, 21]. Early federal reports found that high sickness and death rates in suckling piglets were the main cause of economic loss. This led to fewer animals, lower sow productivity, and breaks in production flow [18]. As the virus spread through major pork-producing areas, these losses quickly became a national economic problem [21].

USDA market outlooks showed clear effects on pork production, with lower output forecasts as PEDV spread during the winter of 2013 to 2014. The Economic Research Service noted that total pork production fell due to piglet losses, but this was partly balanced by heavier slaughter weights and changes in marketing [18]. As a result, the overall drop in annual production was less than piglet mortality numbers alone would suggest, although the pork value chain still faced major economic challenges. Peer-reviewed studies measured both the cost of PEDV and how those costs were distributed. Schulz and Tonsor [18] showed that PEDV acted as a typical supply-impacting disease, with different economic effects across parts of the industry, changing prices and profits for producers, processors, and related businesses. Other welfare-based models estimated that the outbreak reduced U.S. economic welfare by several hundred million dollars to over one billion dollars each year, highlighting its wide economic impact [18].

After major outbreaks, the economic effects of PEDV often go beyond just the immediate production losses. In areas where the virus becomes endemic, producers face ongoing costs for vaccines, better biosecurity, testing, and feed-risk management as part of regular disease control. These repeated costs add to the total economic burden of PEDV, but they are not always included in short-term loss estimates that focus mainly on deaths or sudden drops in production [54, 57].

### Public health impact of PEDV

PEDV does not infect humans and poses no direct zoonotic risk. However, it influences public health by affecting food security, economic stability, and the resilience of agricultural systems. Pork is a key source of animal protein worldwide, so PEDV outbreaks that disrupt pork production can affect food availability and prices, especially in areas that rely heavily on pork or have few other dietary options [18, 57]. When PEDV outbreaks occur on a large scale, they lower the supply of pigs and cause pork prices to fluctuate. These changes hit lower-income groups the hardest, since they spend more of their income on food. Even if these effects are temporary at the national level, they show how animal diseases can affect access to nutrition and fairness in food systems. PEDV is an example of how animal health problems can affect food supply and public health, even if the disease does not infect people directly [18, 57].

PEDV outbreaks put significant mental and financial pressure on farm families and rural communities. Losing many piglets, not knowing how the virus spreads, and the high costs of long-term control measures all add to the stress and lower well-being for producers and agricultural workers. These challenges are similar to those observed in other livestock disease emergencies and underscore the importance of considering social and behavioral factors when assessing the public health effects of animal disease outbreaks [54, 57]. PEDV has shown weaknesses in public health preparedness. Studies have found that the virus can survive for a long time in feed, last in the environment during cold weather, and spread easily through transport networks. This shows how modern food production can facilitate the spread of disease. These results are not just about PEDV; they also offer important lessons for managing new diseases in interconnected animal and environmental systems [11, 27, 54].

PEDV does not directly infect humans, but it can disrupt food systems, put pressure on rural communities, and reveal problems where animal health, the environment, and society meet. Understanding these indirect effects supports the use of One Health strategies that bring together veterinary monitoring, agricultural policy, and public health planning to better prepare for future livestock disease emergencies [54, 57].

### Knowledge gaps and research priorities

Even after more than fifty years of research, PEDV is still not fully controlled. This shows the ongoing gaps in our understanding of viral immunity, evolution, and how the virus spreads. The continued appearance of new genetic variants, along with PEDV becoming a constant problem rather than just occasional outbreaks, suggests that current control methods are not enough to stop long-term transmission in today’s swine production systems [11, 54, 57].

One major question is why it is hard to achieve strong, lasting immunity against PEDV, especially in breeding herds. While natural infection or vaccination can trigger systemic antibody responses, the factors that lead to protective lactogenic mucosal immunity are still not well understood. Both field and laboratory studies show that maternal IgA responses can vary in strength and duration, which indicates that piglets are not always fully protected even if their mothers were previously exposed or vaccinated [10, 16, 57]. It is still unclear how viral changes, immune memory, and host factors each contribute to these problems. This highlights the need for studies that directly connect mucosal immune responses to protection in real-world conditions [57].

The ongoing evolution of PEDV through mutation, recombination, and the appearance of S-INDEL and large-deletion variants adds to these immunologic challenges. Many molecular studies show that field strains often differ antigenically from the classical CV777-derived vaccine strains, which raises concerns about how well current vaccines protect against them [23, 24, 33, 40]. While changes in the spike glycoprotein are known to help the virus evade the immune system, new evidence suggests that changes in other genes, such as ORF3 and N, may also affect disease severity, viral replication, and immune responses. However, we still do not fully understand the importance of these non-spike changes for immunity [3, 14, 45–47]. To improve vaccine updates and evaluation, it is important to find ways to better combine genomic surveillance with immunologic and challenge data [40, 47, 54].

While the main ways PEDV spreads are well known, there are still important questions about how the virus survives between outbreaks. Experiments have shown that infectious PEDV can persist for a long time in feed, manure, and the environment when conditions are right. However, it remains difficult to quantify how much these sources contribute to ongoing outbreaks in real-world settings [11, 27, 54]. Although studies suggest that environmental contamination and spreading during transport lead to recurrent farm outbreaks, more research is needed to connect the detection of the virus in the environment to actual transmission risk [11, 27, 54]. Filling this knowledge gap is important for deciding where to invest in biosecurity and how to improve control strategies [54].

Modern swine production systems are closely connected, but we still do not fully understand how their network structure affects the long-term evolution of PEDV. Studies using network analysis and machine learning show that animal movement is a major factor in predicting outbreak risk. However, it is still unclear how these movement patterns influence the persistence, replacement, and regional spread of viral lineages over time [6, 28, 29]. Combining genomic data with information on animal movement, the environment, and production practices is an important research goal for predicting and stopping ongoing transmission in areas where the disease is common [6, 28, 29].

PEDV is still not widely used as a model to study how coronaviruses persist and adapt in livestock. Because PEDV circulates for long periods, has high genetic flexibility, often recombines with other swine coronaviruses, and resists traditional control methods, it offers a valuable chance to study coronavirus evolution under immune pressure in large-scale farming [3, 47, 57]. Using PEDV as a model could help develop better ways to manage ongoing animal coronavirus infections and prepare for future livestock disease outbreaks [47, 57].

Together, these gaps point to key areas for future research. The possible areas of research include defining strong indicators of mucosal protection in breeding herds, matching vaccine design to up-to-date genomic data, measuring the extent to which environmental reservoirs contribute to disease spread, and using system-level data to better predict and stop virus transmission. Making progress in these areas is crucial for moving PEDV control from reactive responses to outbreaks to the development of long-term, sustainable management strategies [11, 54, 57].

### Control and policy implications

PEDV remains common in major swine-producing areas, and current production methods have not eliminated the virus. Even with better diagnostics, biosecurity, and vaccination, PEDV often returns, evolves, and persists within herds. Because of this, control strategies should shift away from focusing solely on outbreaks and adopt ongoing, system-wide management that considers how the virus evolves, how farms are connected, and the fact that not all animals are immune [11, 54, 57].

Vaccination is still a key avenue for controlling PEDV, but field experience shows there are important challenges. Many commercial vaccines are made from older strains that do not match the viruses currently found in the field, especially G2b and recombinant types. This mismatch leads to uneven protection, particularly against severe disease in newborn pigs, even when sows are vaccinated [23, 24, 40, 57]. Also, because we do not have clear markers of protective mucosal immunity, it is hard to evaluate and update vaccines, which limits the effectiveness of vaccination on its own [16, 57]. These results suggest that relying solely on vaccination is not enough and that vaccine strategies should be guided by up-to-date genomic surveillance [40, 57].

Repeated PEDV outbreaks across different production systems have shown that eradication is unlikely to be achievable under current conditions. Control should therefore be approached as long-term management of an endemic pathogen rather than as an elimination effort. Network-based modeling suggests that focusing control efforts on high-connectivity nodes, such as sow farms, transport hubs, and feed mills, may yield disproportionate benefits in reducing transmission risk and limiting regional persistence [6, 28, 29]. Although PEDV is not zoonotic, its control has broader implications for food-system resilience and One Health preparedness. The virus illustrates how endemic livestock pathogens can exploit environmental persistence, globalized supply chains, and dense production networks to maintain circulation despite control efforts. Combining animal health surveillance with environmental monitoring and planning for agricultural infrastructure will make systems more resilient, not just to PEDV but also to future livestock disease emergencies caused by similar weaknesses [54, 57].

### PEDV as a model coronavirus in livestock systems

PEDV is a valuable but underutilized model for studying coronavirus persistence, evolution, and control in intensive livestock systems. Unlike newly emerging coronaviruses, PEDV has circulated in pigs for over five decades, shifting from sporadic outbreaks to sustained endemic transmission on multiple continents. The long-term presence, genetic diversity, and repeated failure of standard control methods offer important lessons about how coronaviruses adapt under immune pressure in managed animal populations [11, 54, 57]. Researchers have found that PEDV undergoes point mutations, insertions, and deletions in its spike gene, as well as frequent recombination with other swine coronaviruses. These changes have led to several genotypes and sublineages, including the highly virulent G2b strains, S-INDEL variants, and recombinant viruses with distinct antigenic features [23, 24, 33, 40]. Many of these changes seem to happen in herds with only partial or mixed immunity, showing that incomplete protection can drive viral evolution instead of stopping its spread. This helps PEDV survive even after repeated exposure and control attempts [47, 57].

PEDV continues to circulate even with widespread vaccination and biosecurity, showing how difficult it is to control coronaviruses. In practice, vaccines help reduce the severity of the disease but often do not prevent infection or further spread, especially when the vaccine does not match the virus circulating in the field. These challenges reflect common features of coronavirus ecology, such as immune escape, antigenic drift, and recombination, which help the virus persist even when many animals are immune [11, 57]. Because of this, PEDV shows why it may not be possible to fully eliminate many coronaviruses in dense animal populations. PEDV also shows how the virus can survive in the environment and spread indirectly. Studies have found that the virus can stay infectious for a long time in feed, manure, and other materials, and outbreaks have been linked to contaminated vehicles and shared equipment. These results show that coronaviruses can spread not only through direct contact between animals, but also through the environment and shared systems in production settings [11, 27, 54].

The virus spreads in ways that demonstrate how connected modern swine production is. Studies using network analysis and machine learning find that animal movement and links between production systems are the main factors predicting outbreaks. This shows that coronaviruses can use these networks to keep spreading, even if individual farms have strong controls. It also suggests that system-wide factors may be more important than what happens on a single farm for long-term disease patterns [6, 28, 29]. Overall, the ability of PEDV to adapt, survive under immune pressure, remain stable in the environment, and spread through connected production networks makes it a good model for studying coronaviruses in livestock. What we learn from PEDV can help us understand other animal (and perhaps human) coronaviruses and improve how we monitor, vaccinate, and protect animals in large-scale farming. Using PEDV as a model coronavirus suggests we should focus less on eliminating it and more on managing it, taking into consideration on how the virus evolves and how production systems operate [11, 54, 57].

## Discussion

Over fifty years after it was first identified, PEDV still affects swine herds worldwide, despite significant research, vaccination, and biosecurity measures. This ongoing presence highlights the basic challenges of controlling coronaviruses in crowded, connected production systems, rather than just problems with specific interventions. Experience with PEDV shows that even strong control programs struggle to manage viruses that evolve rapidly, fail to trigger full mucosal immunity, and spread through multiple indirect routes [11, 54, 57].

Field studies show that vaccination reduces illness severity and mortality, especially in newborn piglets, but it rarely prevents infection or viral shedding [23, 40, 57]. These problems are most obvious when the vaccine does not match the viruses currently spreading, as seen with the rise and ongoing presence of G2 and recombinant strains [23, 33, 40]. This pattern is common in coronavirus ecology, where immune pressure shapes viral evolution but does not prevent viral spread. Because of antigenic drift and recombination, these viruses continue to circulate in populations that have some, but not complete, immunity. This explains why it is difficult to eliminate many endemic coronaviruses in large, intensive animal populations [11, 33, 57].

Protecting newborn piglets relies on strong lactogenic immunity, but this is one of the most unpredictable parts of PEDV control. While infection or vaccination leads to systemic antibody responses in sows, the amount and persistence of mucosal IgA in milk can differ greatly between herds [10, 16, 57]. As a result, piglet protection is often incomplete, even after repeated exposure or vaccination. Without clear immunologic markers linking sow immunity to piglet outcomes, it is difficult to evaluate and update vaccines, which limits the effectiveness of immunization on its own [57].

Ongoing changes in the virus make these issues even harder to manage. PEDV evolves through point mutations and recombination, especially in the spike gene. However, point mutations and recombination also occur in other regions that affect how the virus replicates, causes disease, and interacts with hosts [3, 14, 45, 46, 57]. It should be realized that genetic similarity does not always imply that the virus will have the same antigens, so using sequence data alone is not sufficient to guide vaccine updates [40, 57]. Control strategies will work better if they combine genomic surveillance with studies of antigenic and phenotypic traits, rather than relying solely on genetic distance to predict immune escape [40, 57].

Besides host immunity and viral evolution, PEDV persists because it stays stable in the environment and spreads indirectly. Research shows the virus can survive for long periods in feed, manure, and other materials. Epidemiologic research also points to contaminated transport vehicles, shared equipment, and production facilities as common sources of disease spread [11, 27, 54]. Evidence shows that PEDV transmission is not limited to direct contact between animals but also relies on environmental sources and connections within production systems to keep circulating in the region [11, 27, 54].

Overall, the evidence supports shifting PEDV control away from eradication toward sustained risk reduction, as the virus continues to evolve and exploit multiple transmission routes. Genomics-informed surveillance, clearer definition of protective immunity in piglets, and targeted interventions at high-impact nodes, such as transport and feed systems, are likely to have the greatest impact [11, 28, 29, 57]. PEDV is not only important for swine health but also serves as a useful example for studying how coronaviruses persist under immune pressure and for developing strong control strategies for other coronaviruses in managed animal populations [11, 54, 57].

## Conclusion

PEDV highlights the ongoing challenges of managing endemic coronaviruses in intensive livestock systems. Even after more than fifty years since it was first identified, PEDV still spreads worldwide. This is due to its ability to change genetically, limited protective immunity, persistence in the environment, and the close connections within modern swine production. This review shows that PEDV persists due to the combined effects of viral evolution and disease spread through production systems, not just because of problems with vaccination or biosecurity [11, 54, 57]. Even with significant progress in understanding PEDV at the molecular level, improving diagnostics, and developing control methods, it remains difficult to achieve lasting protection for entire swine populations. Problems like inconsistent immune responses in sows, differences between vaccine strains and those found in the field, and frequent genetic changes in the virus make long-term control difficult, especially in breeding herds and newborn piglets [16, 23, 40, 57]. These challenges show that we need to shift from fixed, outbreak-focused methods to more flexible strategies that use real-time genomic and epidemiological data [40, 54, 57].

The impact of PEDV goes beyond just production losses. Ongoing outbreaks and their continued presence create lasting financial challenges for producers, disrupt pork supply chains, and reveal weaknesses in feed, transport, and production systems. While PEDV does not pose a risk to humans, the ability to disrupt food systems and affect rural communities makes it important to consider within the One Health approach, which focuses on system resilience and preparedness [18, 54, 57]. PEDV is also a useful example for studying how coronaviruses behave in managed animal populations. The ongoing spread despite immune responses, dependence on environmental and network-based transmission, and the repeated shortcomings of traditional control methods offer lessons for other animal coronaviruses. Learning from PEDV can help improve surveillance, update vaccines, and strengthen biosecurity policies for different species and production systems [11, 54, 57].

PEDV control should focus less on eradication and more on managing the virus as an endemic problem. Effective strategies should combine sustained herd immunity, improved protection of neonatal piglets, and coordinated biosecurity at high-connectivity points such as transport and feed systems, supported by continuous genomic surveillance. Approaches that reflect how the virus actually evolves and move through modern production systems will not only reduce the long-term impact of PEDV but also improve preparedness for future livestock disease events driven by similar system-level vulnerabilities [11, 28, 29, 57].

## Data Availability

No datasets were generated or analysed during the current study.
